# Anatomical Variants of Condylar Process, Coronoid Process, and Sigmoid Notch in a Maharashtrian Population: A Radiographic Study

**DOI:** 10.7759/cureus.40434

**Published:** 2023-06-14

**Authors:** Subharina Mahapatra, Manjula Hebbale, Amit Mhapuskar, Rajshekhar Halli, Ananya Mittal

**Affiliations:** 1 Oral Medicine & Radiology, Bharati Vidyapeeth (Deemed to be University) Dental College & Hospital, Pune, IND; 2 Oral and Maxillofacial Surgery, Bharati Vidyapeeth (Deemed to be University) Dental College & Hospital, Pune, IND

**Keywords:** orthopantomography, human identification, sigmoid notch, condyle, coronoid process

## Abstract

Background

The cornerstone of an individual’s singularity is identification. Digital orthopantomography (OPG) helps to illustrate the varying condylar process, coronoid processes, and sigmoid notch found within a population to facilitate individual recognition. This study aims to assess the various shapes of the condylar process, coronoid process, and sigmoid notch found using OPG in a sample population of an Indian city.

Methodology

This retrospective, cross-sectional study was conducted using 1,000 good-quality digital OPG scans to evaluate the different shapes. The scans were evaluated by two experienced oral radiologists and tabulated for statistical analysis.

Results

The current investigation revealed varied morphological forms of the three entities, with the round shape being the most frequently observed condylar process, coronoid process, and sigmoid notch. Comparisons across sides and between sexes revealed differences in all three variables which were found to be statistically significant. We discovered a crooked finger condyle (58.56% on the left side and 41.44% on the right side), a beak-shaped coronoid process (50.0% on the left side and 50.0% on the right side), and a V-shaped sigmoid notch (41.35% on the left side and 58.65% on the right side) in this study. This is a unique finding not reported by other studies.

Conclusions

Analyzing the shape of the condylar process, coronoid process, and sigmoid notch found on an OPG scan can help with gender identification in forensic odontology and anthropology as these anatomical features show strong sexual dimorphism.

## Introduction

The human lower jaw, also known as the mandible, is the largest bone in the skull. It also contributes to the process of digestion and helps form the lower jawline. Finally, it maintains the position of the lower teeth. The mandible is the lower jawbone located below the maxilla. It is made up of the body and the ramus. It constitutes the condylar process and coronoid processes. The ramus of the mandible bone contains the coronoid process. As a triangular bone plate, the coronoid process protrudes upward and slightly forward. The variations in the shape of the coronoid process may serve as anthropological markers to evaluate various populations and races. The shape of the sigmoid notch is determined by the shape of the condylar process and the coronoid process [1,2]. Various legal and personal matters pertaining to an individual’s identity require the individual’s identification, including death certification. Identification of a person has been aided by fingerprint analysis and DNA matching for many years [3]. Identification often relies heavily on dental studies and anthropological studies [4,5]. Anthropological and forensic studies rely heavily on the documentation of morphological variations of the condylar process, coronoid process, and sigmoid notch [6,7]. Both the individual’s genetic makeup and the functional changes that accompany maturation contribute to these variations. In forensic dentistry, radiographs play a crucial role in revealing details that would otherwise go undetected by a visual inspection alone. Maxillofacial radiography with an orthopantomography (OPG) is a less expensive method and is used routinely as a screening tool [8].

Hence, this study was conducted to illustrate the morphological variations of the condylar process, coronoid process, and sigmoid notch in gender determination and individual identification using panoramic radiographs.

## Materials and methods

This retrospective study was conducted in the Department of Oral Medicine and Radiology, Bharati Vidyapeeth Dental College & Hospital, Maharashtra, India. Digital OPGs retrieved from the archives of the Department of Oral Medicine and Radiology were used in this study. Carestream machines and PSP sensors (Digora) were used to acquire all digital OPGs, which were then processed in Digora software using the industry-standard exposure parameters. Printouts of these pictures were obtained, and with the aid of the viewer, box tracing was done over projection sheets. All patient identities and the reason for the scan’s diagnosis were concealed to protect the doctor-patient privilege.

This study primarily aimed to evaluate the prevalence of various shapes of condylar process, coronoid process, and sigmoid notch in OPGs. The objectives of this study were to compare the right and left sides of the condyle, coronoid, and sigmoid notch as well as to compare the shape in male and female patients.

The ratio of male-to-female scans was equal. Using the Epi Info software version 7.1 (CDC, USA), the sample size was ultimately determined to be 1,000 using the formula N = Z2PQ/d2.

The current study included 1,000 scans that fulfilled the inclusion and exclusion criteria. Good-quality OPG scans were included in this study. Scans containing errors with partially reconstructed images and artifacts, scans with any pathology or fracture, scans with any bony changes, and scans of patients who had undergone surgery in the sinonasal region were excluded from this study.

**Figure 1:**
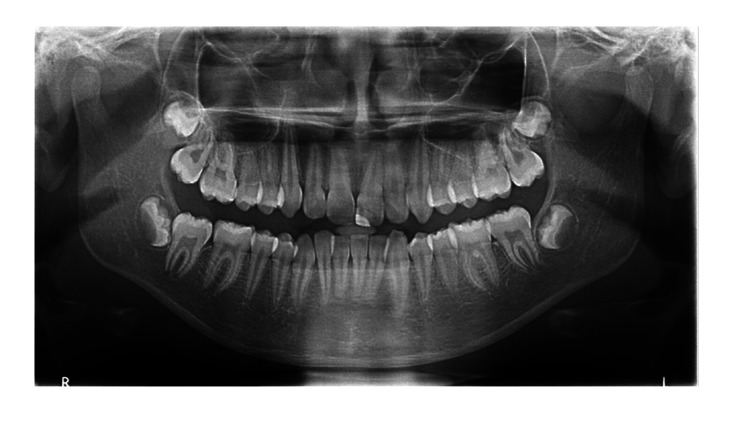
Ideal digital orthopantomogram.

**Figure 2:**
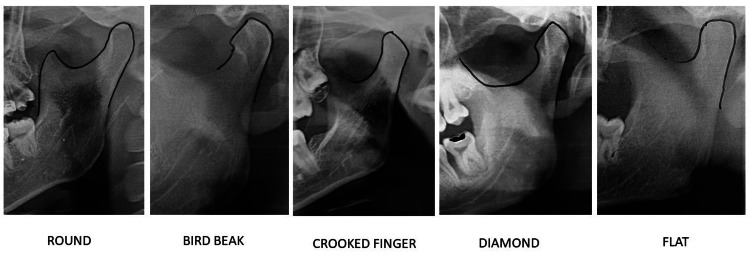
Condylar process.

**Figure 3:**
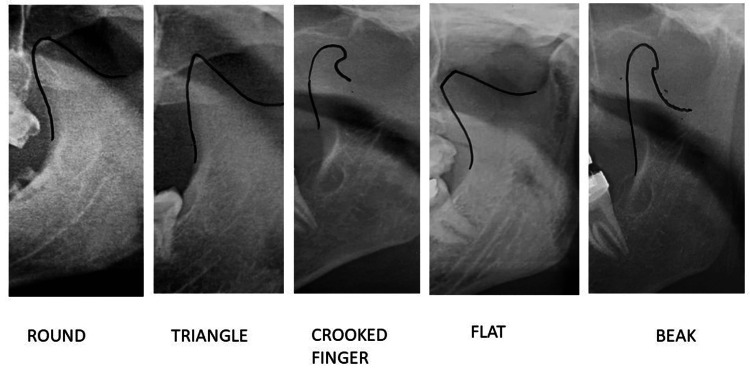
Coronoid process.

**Figure 4:**
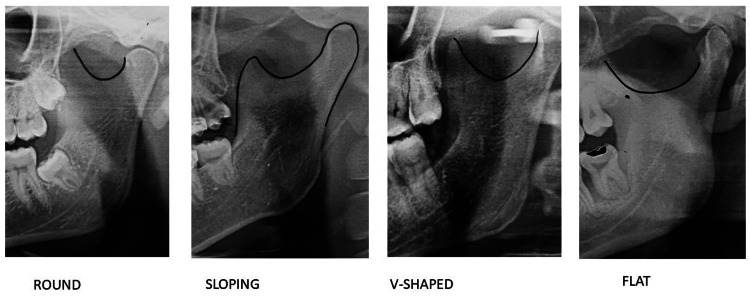
Sigmoid notch.

The shape of the condyle process, coronoid process, and sigmoid notch were interpreted according to Sahithi et al. [5]. The radiographs were used to evaluate the various morphological forms of the condylar process, coronoid process, and sigmoid notch (Figures 1-4).

The data were entered, coded, and checked. Subsequently, an Excel spreadsheet was used to compile the data. SPSS version 24.0 (IBM Corp., Armonk, NY USA) was used for all statistical analyses. Numbers and percentages were used to describe the data, and a chi-square test was performed.

Two experienced oral and maxillofacial radiologists analyzed and interpreted all scans. Each scan was interpreted independently by each observer, followed by a discussion of each radiograph by two observers. Interobserver variability was also performed using kappa statistics on both sides which showed a higher agreement for the condylar process, followed by the coronoid process and sigmoid notch.

## Results

The condylar process was most frequently observed to be round (49.69% on the left side and 50.31% on the right side), followed by crooked finger-shaped (58.56% on the left side and 41.44% on the right side), bird beak (43.55% on the left side and 56.45% on the right side), diamond-shaped (44.92% on the left side and 55.08% on the right side), and flat-shaped. The crooked finger-shaped condyle was the most recently observed and was more common on the left side. Table 1 displays the statistically significant (p = 0.0003) right- and left-sided differences in the shape distribution of the condylar process.

**Table 1:** Comparison of the shape of the condyle.

	Right side		Left side		Chi-square	P-value
Shape	Number	Percentage	Number	Percentage		
Round	501	50.31	495	49.69	20.99	0.0003
Diamond	130	55.08	106	44.92		
Bird beak	153	56.45	118	43.55		
Crooked finger	179	41.44	253	58.56		
Flat	37	56.92	28	43.08		

The coronoid process was most often found to be round (47.35% on the left side and 52.65% on the right side), followed by triangular-shaped (50.43% on the left side and 49.57% on the right side), flat-shaped (57.98% on the left side and 42.02% on the right side), and crooked finger-shaped (60.46% on the left side and 39.54% on the right side and). The beak-shaped coronoid process was the most recently observed shape of the coronoid process, which was equally distributed on both sides. As shown in Table 2, there was a statistically significant difference (p = 0.015) between the right and left sides of the coronoid process distribution.

**Table 2:** Comparison of the shape of the coronoid process.

	Right side		Left side		Chi-square	P-value
Shape	Number	Percentage	Number	Percentage		
Triangular	290	49.57	295	50.43	12.22	0.015
Round	587	52.65	528	47.35		
Beak	1	50	1	50		
Flat	71	42.02	98	57.98		
Crooked finger	51	59.54	78	60.46		

The sigmoid notch was most frequently observed to be round (47.11% on the left side and 52.89% on the right side), followed by wide-shaped (56.7% on the left side and 43.3% on the right side and), sloping-shaped (54.8% on the left side and 45.12% on the right side), and V-shaped (41.3 % on the left side and 58.65% on the right side and). The V-shaped sigmoid notch was the most recently observed shape and the prevalence was more on the right side. Table 3 shows that there was a statistically significant difference (p = 0.00006) in the sigmoid notch shape distribution between the right and left sides.

**Table 3:** Comparison of the shape of the sigmoid notch.

	Right side		Left side		Chi-square	P-value
Shape	Number	Percentage	Number	Percentage		
Round	502	52.89	447	47.11	21.8	0.00006
Sloping	162	45.12	197	54.88		
Wide	197	43.3	258	56.7		
V-shape	139	58.65	98	41.35		

The condylar process was found to be round most of the time in both males and females (51.2% on the left side and 48.4% on the right side in males, and 47.8% on the left side and 50.8% on the right side in females). In male patients, the round shape was followed by a bird beak shape (16.6% on the left side and 17.2% on the right side), then a crooked finger (21.2% on the left side and 14.4% on the right side), a diamond shape (8.02% on the left side and 11.2% on the right side), and flat shape (2.8% on the left side and 5.2% on the right side). In females, the round shape was the most common (47.8% on the left side and 50.8% on the right side), followed by crooked finger (29.4% on the left side and 21.4% on the right side), diamond (13.0% on the left side and 14.8% on the right side), bird beak (7.0% on the left side and 11.4% on the right side), flat (2.8% on the left side and 1.6% on the right side). In our study, the round-shaped condylar process was more common on the left side of males and the right side of females. There was a statistically significant difference (p = 0.0001) between the genders in terms of the shape of the condylar process, as shown in Table 4.

**Table 4:** Comparison of the shape of the condyle between males and females.

	Male						Female					
	Right side		Left side		Chi-square	P-value	Right side		Left side		Chi-square	P-value
Shape	Number	Percentage	Number	Percentage			Number	Percentage	Number	Percentage		
Round	247	48.4	256	51.2	20.09	0.0001	254	50.8	239	47.8	20.89	0.0001
Diamond	56	11.2	41	8.02			74	14.8	65	13.0		
Bird beak	96	17.2	83	16.6			57	11.4	35	7.0		
Crooked finger	72	14.4	106	21.2			107	21.4	147	29.4		
Flat	29	5.2	14	2.8			8	1.6	14	2.8		

In both sexes, a round coronoid process was the most common (54.2% on the left side and 61.4% on the right side in males, and 51.4% on the left side and 56.0% on the right side in females), followed by a triangular shape (28.4% on the left side and 26.8% on the right side), a flat shape (10% on the left side and 7.6% on the right side), a crooked finger (7.4% on the left side and 4.2% on the right side), and a beak shape (0.2% on the left side and 0% on the right side). For female patients, the most common shape was the round shape, followed by triangular (30.6% on the left side and 31.2% on the right side), then flat (9.6% on the left side and 7.6% on the right side), crooked finger (8.2% on the left side and 6.0% on the right side), and beak shape (0.2% on both sides). In our study, the round-shaped coronoid process was more common on the right side for both genders but the triangular-shaped coronoid process was more common on the left side of males and the right side of females. There was a statistically significant difference (p = 0.0001) between the genders in terms of the shape of the coronoid process, as shown in Table 5.

**Table 5:** Comparison of the shape of the coronoid process between males and females.

	Male						Female					
	Right side		Left side		Chi-square	P-value	Right side		Left side		Chi-square	P-value
Shape	Number	Percentage	Number	Percentage			Number	Percentage	Number	Percentage		
Triangular	134	26.8	142	28.4	22.85	0.0001	156	31.2	153	30.6	22.86	0.0001
Round	307	61.4	271	54.2			280	56.0	257	51.4		
Beak	0	0.0	1	0.2			1	0.2	1	0.2		
Flat	38	7.6	50	10			38	7.6	48	9.6		
Crooked finger	21	4.2	37	7.4			30	6.0	41	8.2		

The sigmoid notch was most commonly found to be round shaped (52.2% of males having a right-sided round sigmoid notch and 47.2% having a left-sided notch, and 48.2% of females having a right-sided notch and 42.2% having a left-sided notch). The most common shape of the sigmoid notch in male patients was round, followed by wide (26.8% on the left side and 18.4% on the right side), sloping (17.0% on the left side and 15.8% on the right side), and V-shaped (9.0% on the left side and 13.6% on the right side). In females, the most common shape was the round shape, followed by sloping (22.45% on the left side and 16.6% on the right side), V-shaped (10.6% on the left side and 21.00% on the right side), or wide (24.81% on the left side and 14.20% on the right side). In our study, the round-shaped sigmoid notch was more common on the right side for both genders but the most recently observed shape, a V-shaped sigmoid notch, was more common in females than males on the right side than the left side. There was a statistically significant difference (p = 0.0001) between the genders in terms of the shape of the sigmoid notch, as shown in Table 6.

**Table 6:** Comparison of the shape of the sigmoid notch between males and females.

	Male						Female					
	Right side		Left side		Chi-square	P-value	Right side		Left side		Chi-square	P-value
Shape	Number	Percentage	Number	Percentage			Number	Percentage	Number	Percentage		
Round	261	52.2	236	47.2	22.88	0.00001	241	4.8.2	211	42.2	22.85	0.00001
Sloping	79	15.8	85	17.0			83	16.6	112	22.4		
Wide	92	18.4	134	26.8			71	14.2	124	24.8		
V-shape	68	13.6	45	9.0			105	21.0	53	10.6		

## Discussion

The cornerstone of an individual’s singularity is identification. According to Acharya and Taylor, “The characteristics by which a person may be recognized” [1]. Dental forensic identifications, which are based on a comparison of antemortem and postmortem radiographs, are reliable and effective. Most often, a digital panoramic radiograph or OPG is employed because of its ease of use, broader scope of examination, and investigation of a large area. Disparities in development due to genetic factors or differences in function during growth give rise to observable morphological differences in anatomic structures. The mandible is the largest and most powerful bone in the face. It features a convex forward horizontally curved body and two wide rami ascending posteriorly. The coronoid and condylar processes are found on the rami. Digital OPG scans were used to evaluate the coronoid process, condyle process, and sigmoid notch in this study [6].

This study found that the round-shaped condylar process was the most common among both males and females, followed by beaks, crooked fingers, diamonds, and flats for men. For females, the round shape was the most common, followed by the crooked finger shape, diamond shape, beak shape, and flat shape. Sahithi et al. [5] found that the condyle shape most commonly observed in males was angled, followed by round, and angled in females, which was similar to our study. The round shape is common in both sexes, as shown in other studies conducted by Ribeiro et al. [8] and Choudhary et al. [9] among the Brazilian and East Indian populations, respectively. Our findings are consistent with those of a previous study reported by Oliveira et al. [10] which found that spherical shapes were more common.

In our study of the Maharashtrian population, we found that round coronoid processes were the most common, followed by triangular, flat, crooked finger, and bird beak shapes. Our findings are counter to studies done among the south Indian population by Shakya et al. [6], Sudha et al. [11], and Pradhan et al. [12], who found that triangular shapes were the most common, followed by rounds, beaks, and flats, as well as those done on the north Indian population by Tapas et al. [13] and the south Indian population by Sahithi et al. [5]. In their research on west Indians, Kadam et al. [14] noted that beak shapes were more frequently observed than triangle shapes. Prajapati et al. [15] conducted a study using dry mandibles and the results showed that triangular coronoid processes were commonly followed by round and hook shapes. Isaac et al. [1] found that triangular shapes were the most common, followed by hook and round shapes. Round shapes were almost as common in both men and women, which is in line with the results of this study.

In our study, the round shape of the sigmoid notch was frequently observed among males and females. In males, the round shape was the most common, followed by wide, sloping, and V-shape. In females, the round shape was the most common, followed by sloping, V-shape, and wide. In the study by Shakya et al. [6], the sloping shape was the most common, followed by the round and wide shapes. In a study of south Indians, Sahithi et al. [5] discovered that the most common shape of the sigmoid notch was wide in both sexes. Their observations contradicted our findings.

However, in our study, the variation of all three entities was found to be statistically significant when compared on either side and across both sexes. In this study, we discovered crooked finger condyle, beak-shaped coronoid, and V-shaped sigmoid notch, which is a novel finding. Our findings indicate that using panoramic images to depict various shapes can be used as a much easier and faster method of identifying an individual, particularly in mass disasters where antemortem records are preserved.

## Conclusions

The process of identifying humans through radiography has become extremely important in light of the most recent situation. This is primarily attributable to the procedure’s usability. In our study, the round-shaped condylar process was more common on the left side of males and the right side of females. The round-shaped coronoid process was more common on the right side for both genders, but the triangular-shaped coronoid process was more common on the left side of males and the right side of females. The round-shaped sigmoid notch was more common on the right side for both genders, but the most recently observed shape, a V-shaped sigmoid notch, was more common in females than males on the right side than on the left side. Within the parameters of this study, a wide variety of morphological forms of the condyle, coronoid process, and sigmoid notch were observed; therefore, we conclude that the different shapes can be used as support for gender identification in forensic dentistry.
